# Biodegradation potential of *Gordonia* spp. on polypropylene and polystyrene: enhanced degradation through pretreatment

**DOI:** 10.3389/fmicb.2025.1621498

**Published:** 2025-07-25

**Authors:** Yan Zhu, Hongzhe Wang, Jing Bai, Yanjie Qi, Dongfei Han

**Affiliations:** ^1^School of Environmental Science and Engineering, Suzhou University of Science and Technology, Suzhou, China; ^2^Key Laboratory of Soil Environment and Pollution Remediation, Institute of Soil Science, Chinese Academy of Sciences, Nanjing, China; ^3^School of Chemistry and Life Sciences, Suzhou University of Science and Technology, Suzhou, China

**Keywords:** *Gordonia* spp., polypropylene, polystyrene, biodegradation, ATR-FTIR, genomic analysis

## Abstract

**Introduction:**

As extensively utilized synthetic polymers, polypropylene (PP) and polystyrene (PS) have raised significant environmental concerns due to their persistent accumulation in ecosystems.

**Methods:**

To enhance biodegradation efficiency, we implemented a dual pretreatment approach combining thermal activation and fenton’s reagent oxidation prior to microbial treatment. Through a systematic 50-day incubation experiment with single-strain cultures of five *Gordonia* strains (*Gordonia polyisoprenivorans* B251, *Gordonia polyisoprenivorans* B253, *Gordonia hydrophobica* 4.134, *Gordonia humi* 4.135, and *Gordonia sihwensis* LQ21), we quantitatively evaluated the degradation performance using four complementary analytical methods: mass loss quantification, attenuated total reflectance Fourier transform infrared spectroscopy (ATR-FTIR), and scanning electron microscopy (SEM) and surface water contact angle.

**Results:**

Notably, *Gordonia polyisoprenivorans* B253 demonstrated remarkable degradation capabilities, as evidenced by: (1) characteristic chemical modifications including hydroxyl group formation (3,280 cm^−1^), carbon-carbon double bond generation (1,640 cm^−1^), and ether group appearance (1,100 cm^−1^) in ATR-FTIR spectra; (2) pronounced surface erosion patterns observed via SEM; and (3) significant mass reduction (1.927% ± 0.038% of PS) compared to controls. Comparative analysis revealed that combined thermal-fenton pretreatment enhanced biodegradation efficiency about by 1.3-fold compared to untreated samples, suggesting synergistic effects between physicochemical pretreatment and biological degradation. Genomic characterization of B253 identified putative catabolic enzymes, including alkane hydroxylases, cytochrome P450 systems, alcohol-dehydrogenase, styrene monooxygenase and epoxide hydrolase potentially responsible for polymer breakdown.

**Discussion:**

This work advances plastic biodegradation by identifying novel PP/PS-degrading *Gordonia* species, establishing an effective pretreatment protocol, and providing genomic insights into biodegradation pathways. These findings contribute to developing sustainable solutions for managing persistent plastic waste. These products or metabolites from the degradation of PP and PS plastics can be further extracted and processed into new plastic raw materials or other valuable products, facilitating the recycling of plastic resources. This approach not only decreases reliance on fossil resources, but also mitigates energy consumption and carbon emissions during of plastics production. Thus, it promotes the development of green and sustainable plastics industry and contributes to the establishment of a circular economy. Furthermore, we believe there is also great potential for addressing plastic pollution through various integrated treatment methods.

## Graphical Abstract

Diagrams of experimental setup and results. The left section shows the sample of particles of polypropylene and polystyrene, which were then pretreated with heat and fenton reactions. The center describes a reaction mixture in which *Gordonia* spp. is added to polypropylene or polystyrene and incubated in a vessel. The right section shows the results of the experiment, including bar graphs, microscopic images, and data graphs, indicating the effect of the treatment. 
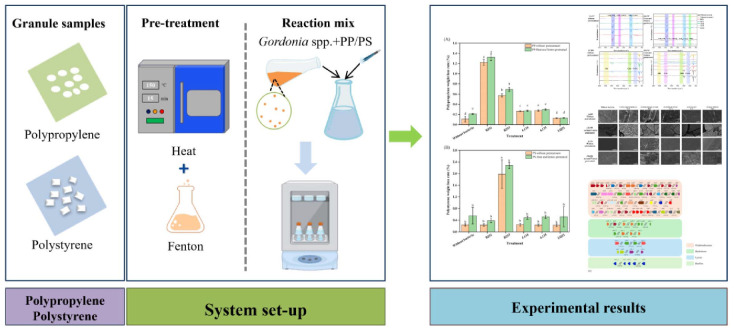

## 1 Introduction

Plastic is a material made of polymers (usually synthetics) that are malleable and changeable. Plastic as unmanaged waste is a huge environmental problem (Xanthos and Walker, 2017; Wayman and Niemann, 2021), and many plastic products turn into rubbish after use, which is difficult to degrade and causes environmental pollution. In addition, some plastic products may contain hazardous substances, which can affect human health in long-term use. It is estimated that about 0.1% of the plastics produced globally to 4.1% (Jambeck et al., 2015) ends up in the ocean in the form of so-called marine plastic debris. Of these, polyethylene (PE) is the most produced plastic polymer, accounting for about 30% of total plastic production, and is one of the most corrosion-resistant polymers, along with polypropylene (PP) and polystyrene (PS), which account for 70% of total production. This high corrosion resistance is at the root of the plastic waste problem and the accumulation of discarded plastic materials and items (Bertocchini and Arias, 2023).

Plastics usually consist of polymers containing a large number of carbon-carbon and carbon-hydrogen bonds, which makes them difficult to degrade by microorganisms (Liu L. C. et al., 2022). Microorganisms degrade organic compounds by producing specific enzymes, but for the carbon-carbon and carbon-hydrogen bonds in plastics, they lack enzymes that can effectively degrade these bonds. In contrast, microorganisms are more capable of degrading natural organic matter found in nature because they have evolved degrading enzymes adapted to these organisms (Chen et al., 2020; Sheridan et al., 2022; Kothawale et al., 2023). Although some microorganisms are capable of degrading plastics to some extent, their degradation rate is usually slow. This means that even if there are microorganisms that are able to degrade plastics, they will take a long time to fully degrade plastic products, which is not realistic for alleviating the plastic pollution problem (Zeenat et al., 2021). The use of microorganisms and the enzymes they secrete to degrade plastics has the advantages of being environmentally friendly, substrate specific and sustainable, with most enzymes operating at ambient temperatures and neutral pH, which reduces energy consumption (Wei and Zimmermann, 2017), and is therefore seen as a highly promising solution. For example, the efficient depolymerisation of PET plastics by *Ideonella sakaiensis* and the hydrolysis of polyester-based plastics by keratinases are important examples of biodegradation technologies. Enzymatic degradation does not require high temperatures and pressures or strong chemicals, which reduces secondary contamination (Yoshida et al., 2016; Othman et al., 2021), and degradation products (e.g., monomers) can be recycled and reused (Tournier et al., 2020). Degradation products (e.g., TPA, EG) can be used directly in the synthesis of new plastics (Ellis et al., 2021; Lai et al., 2023). However, for inert plastics with C-C backbone structures such as polyethylene (PE), polypropylene (PP) and polystyrene (PS), the degradation efficiencies of these methods are usually insufficient (typically below 20%) and the degradation cycles are months long. The main reason for this limitation is that these plastics are highly hydrophobic, highly crystalline and have large molecular weights, which make it difficult for microorganisms to directly access and cleave the polymer chains (Kawai et al., 2020). The oxidation of polyolefins can be monitored using various analytical methods, including thermal analysis via differential scanning calorimetry, rheology, tensile properties, and excitation/vibration spectroscopy. One of the most used techniques for tracking oxidation reactions is Fourier-transform infrared (FTIR) spectroscopy, which facilitates the observation of changes in the carbonyl bond and has led to the development of carbonyl index (CI). By detecting functional groups across different spectral bands, FTIR analysis can also monitor other chemical changes that occur throughout the life cycle of a material (Mellor et al., 1973; Almond et al., 2020; Mitrano and Wagner, 2022). Therefore, efficient pre-treatment technologies can increase the rate of microbial degradation of plastics and are key to breaking the current bottleneck.

Pretreatment techniques are a preliminary step before biological methods are considered. In most previous studies, various pretreatments have been undergone prior to the biodegradation step, which is essential to reduce the hydrophobicity of the polymer, making it vulnerable to microbial attack. The pretreatment techniques employed include UV, γ irradiation, heat, and fenton treatment (Samat et al., 2023), and the introduction of C = O or –OH into the C-C backbone of PP will result in easy degradation of the polymer. Based on the weight loss and chemical structure analysis of the polymer, UV and heat-treated PP samples are more susceptible to degradation than untreated samples. After mechanical crushing, the specific surface area of PE film increased by 50%, and the degradation rate in compost increased from 2 to 12% (Shah et al., 2008). Compared to UV-treated PP films, fenton-treated films had more microorganisms attached after 12 months, and PP pretreated with short UV light became more hydrophilic and also had a significant weight reduction of 2.5% (Arkatkar et al., 2010). The weight loss rate of PET treated with 5% NaOH increased from 1 to 20% within 8 weeks (Yoshida et al., 2016). Two novel reactions, the bio-fenton reaction using glucose oxidase alone and the bio-light-fenton reaction using GO_*X*_ immobilized on titanium dioxide nanoparticles under ultraviolet radiation, were tested, and gas chromatography-mass spectrometry (GC-MS) analysis found that small organic acids such as acetic acid and butyric acid were the main metabolites released by sulfonated PE (SPE) (Ghatge et al., 2022).

The genus *Gordonia* is a gram-positive bacterium of the order *Actinomycetes* and contains mycolic acid in its genome with a high G/C value. It is ecologically involved in the carbon cycle of organic matter in soil and water due to the characteristics of environmental biotechnology and bioremediation, especially in the degradation of persistent organic compounds (Arenskötter et al., 2004). It is well documented that *Gordonia* spp. have the ability to degrade organic compounds of various molecular structures (Drzyzga, 2012). In previous studies, alkane degradation was a common feature of the genus (Kummer et al., 1999; Xue et al., 2003; Kotani et al., 2007; Lo Piccolo et al., 2011). A number of *Gordonia* sp. strains have been characterized as having the ability to biodegrade polymers and alkane compounds such as synthetic rubber (e.g., *cis*-1,4-polyisoprene), petroleum, diesel and styrene (Arenskötter et al., 2004; Hiessl et al., 2012; Heine et al., 2018; Trögl et al., 2018; Silva et al., 2019). However, a number of enzymes related to hydrocarbon biocatalysis have been identified in the genome of *Gordonia* spp. (Lienkamp et al., 2021). In addition to direct catalytic capacity, the production of extracellular acidic polysaccharides and biosurfactants by *Gordonia* sp. has been shown to induce the formation of cellular aggregates (Kondo et al., 2000) and to enhance substrate bio-accessibility and cellular uptake (Arenskötter et al., 2004; Trögl et al., 2018). In our previous study, we isolated a strain, *Gordonia polyisoprenivorans* B251 from landfill that degraded polyethylene (Wang et al., 2023). *Gordonia* spp. degrades C-C bonded polymers, it secretes oxidative enzymes (e.g., *Lcp*) that are remarkably efficient in degrading double bonded polymers (natural rubber) (Hiessl et al., 2012; Lynd et al., 2022; Walker et al., 2023; Stoett et al., 2024). In mixed communities, *Gordonia* spp. can break down long-chain polymers into smaller molecules and then further processed by other microorganisms. (Liu et al., 2023). Recently, a polystyrene-degrading bacterium, *Gordonia* sp. PS3, was isolated from the intestinal tract of murine gall larvae. The degradation rate of PS-microplastics (PS-MPs) by this strain was found to 33.59 ± 1.12% after 40 days (Xu et al., 2025). However, previous studies have not report any evidence of *Gordonia* spp. degrading polypropylene (PP).

To order to address the inefficiency of *Gordonia* spp. in degrading C-C plastics and to clarifying the degradation mechanism, five strains of *Gordonia* spp. were utilized as strains to assess their ability to degrade polypropylene and polystyrene. Additionally, a thermo-chemical fenton pretreatment system was developed to investigate the enhanced biodegradationprocess of pretreatment. After a 50-day incubation cycle, a strain, *Gordonia polyisoprenivorans* B253, was found to be capable of degrading polypropylene and polystyrene particles. The degradation activity of the strain will be characterized by the loss of weight of the plastics, changes in the chemical structure (appearance of oxygen-containing groups) and the surface morphology of the particles, and the pathways will be predicted through the analysis of the genome.

## 2 Materials and methods

### 2.1 Polymer materials and culture media

Polypropylene particles (melt flow index 12 g/10 min, Mn ∼42.08) were acquired from Maclean’s Reagent. Polystyrene pellets (approximately 3 mm in particle size) were obtained from Sino-Singapore Plastics Company.

Luria-Bertani (LB) medium contains 10 g/L tryptone, 5 g/L yeast extract and 10 g/L NaCl as a liquid nutrient broth medium. Liquid basal medium (MM, pH 7.2∼7.4) with 1% (w/v) polypropylene and polystyrene as the sole carbon source was used as enrichment medium, which contained (per 1,000 mL) 1.0 g KH_2_PO_4_, 6.0 g K_2_HPO_4_, 0.2 g MgSO_4_⋅7H_2_O, 1.337 g NH_4_Cl, 1.189 g KNO_3_, 0.5 g NaCl and 10 mL of trace elements.

### 2.2 Pre-treatment of polypropylene and polystyrene

Samples were labeled as PP granule (PP-without pretreatment), heat-treated and fenton-treated PP granule (PP-Heat and fenton pretreated), PS granule (PS-without pretreatment), heat-treated and fenton-treated PS granule (PS-Heat and fenton pretreated). Two different pretreatment overlay strategies were used for PP and PS samples. Heat treatment involved the treatment of samples in an oven 150°C for 15 min. For the Fenton treatment of plastics, specifically, 0.305 mL of 30% H_2_O_2_ and 19.4 mL of pure water were added to a vial and mixed, followed by the sequential addition of 0.3 mL of 200 mM Fe^2+^ (FeSO_4_⋅7H_2_O) and heated-pretreated polypropylene and polystyrene plastics (Liu et al., 2019; Ghatge et al., 2022). The pH of the solution was pre-adjusted to 4.0 with 6 mol/L hydrochloric acid, and the same amount of Fe^2+^ and H_2_O_2_ was added to the mixed solution every 12 h to enhance the oxidation of the plastics, and the solution was changed every 2 days. Control samples contained only plastic and ultrapure water and were incubated on a rotary shaker at 190 rpm for 7 days (Liu et al., 2019; Liu B. et al., 2022; Samat et al., 2023). The effect of pretreatment on the degradation of plastics by the strain was explored in a combination of the two treatments.

### 2.3 Cultivation and identification of *Gordonia* strains

Revived culture of *Gordonia* strains (the details of these strains are presented in Table 1) on solid medium were transferred to LB liquid medium, and incubated at 28°C with shaking at 120 rpm for 2 days (Wang et al., 2023). 1∼2 mL of the bacterial solution was collected into a centrifuge tube and centrifuged at 10,000 rpm for 1 min, the supernatant was aspirated, and the genomic DNA of the strain was extracted according to the instructions of the kit. The concentration of genomic DNA was determined using the ScanDrop Micronuclei Acid Protein Detection Spectrometer. The concentration ratio (OD_260_/OD_280_) is generally 1.7∼1.9. A low or high-ratio can indicates that the DNA extraction is not pure. The taxonomy of bacterial were identified by amplification of 16S rRNA gene the universal primers 27F (5′-AGAGTTTGATCCTGGCTCAG-3′) and 1492R (5′-GGTTACCTTGTTACGACTT-3′). PCR amplification was performed as follows: Initial denaturation at 95°C for 5 min; Denaturation at 95°C for 30 s, annealing at 58°C for 30 s, and extension at 72°C for 1.5 min; and extend for a final extension at 72°C for 10 min. The obtained sequences were submitted to the National Centre for Biotechnology Information (NCBI) GenBank database and aligned using the search tool Basic Local Alignment Search Tool (BLAST).^1^ Finally, all cultures were stored in 40% glycerol (v/v) at –80°C for subsequent experiments.

**TABLE 1:** Species information for five strains.

Strain	Origin	Condition of culture	Collection	Deposit number
*Gordonia polyisoprenivorans* B251	Plastic-contaminated soil	LB medium 28°C	China General Microbiological Culture Collection Center (CGMCC)	CGMCC No.23920
*Gordonia polyisoprenivorans* B253	Plastic-contaminated soil	LB medium 28°C	NA	NA
*Gordonia sihwensis* LQ21	Donated by Dr. Zhiyong Ruan	LB medium 28°C	NA	NA
*Gordonia hydrophobica* 4.134^T^	Biofilter composting	LB medium 28°C	Guangdong Microbial Culture Collection Center (GDMCC)	CGMCC No.4.134
*Gordonia humi* 4.135^T^	Soil	LB medium 28°C	Guangdong Microbial Culture Collection Center (GDMCC)	CGMCC No.4.135

“NA” means unknown.

### 2.4 Experimental design of PP and PS treatment

To investigate the degradation of polypropylene and polystyrene by *Gordonia* spp. and to assess whether pretreatment of plastics enhances degradation, experiments were designed (Table 2). Each treatment was conducted in triplicate. Polypropylene and polystyrene were added to inorganic salt medium without carbon source (MM medium) and incubated separately, and the growth curves of each strain were plotted before determining the incubation time.

**TABLE 2:** Polypropylene/polystyrene degradation protocol table.

Plastic handling conditions	Biological treatment conditions	Experiment description
Without pretreatment	Without bacteria	PP/PS_without pretreatment + without bacteria
	*Gordonia* spp.	PP/PS_without pretreatment + bacteria
Heat and fenton pretreated	Without bacteria	PP/PS_Heat and fenton pretreated + without bacteria
	*Gordonia* spp.	PP/PS_Heat and fenton pretreated + bacteria

### 2.5 The gravimetric weight loss of polypropylene and polystyrene

After incubation with microorganisms at 28°C, PP and PS particles were recovered, respectively, and use 2% SDS for 8 h to remove surface biofilm and impurities by ultrasound machine. Wash the samples three times with a 70% alcohol solution and finally three times with sterile water. Place the pellets on an ultra-clean table overnight to air dry. The percentage of mass loss of plastic was calculated using the following formula (Mohan et al., 2016; Liu et al., 2023).


(1) $$Percentageofmassloss\left(\%\right)=\frac{i\,n\,i\,t\,i\,a\,l\,w\,e\,i\,g\,h\,t-f\,i\,n\,i\,a\,l\,w\,e\,i\,g\,h\,t}{i\,n\,i\,t\,i\,a\,l\,w\,e\,i\,g\,h\,t}\times 100\%$$

### 2.6 Fourier-transform infrared spectroscopy (FTIR) analysis

ATR-FTIR spectroscopy (BRUNI TENSOR II infrared spectrometer, Germany) was used to analyze the structural changes of PP-without pretreatment, PP-Heat and fenton pretreated, PS-without pretreatment, PS-Heat and fenton pretreated samples during mixed and un-inoculated strains in the frequency range of 4,000–400 cm^–1^. During degradation, the formation or disappearance of carbonyl groups and methyl groups on methylene groups is monitored. The carbonyl index measures the concentration of the carbonyl group, and the double bond index measures the concentration of the double bond in the plastic. In addition, the hydroxyl index measures the degree of oxidation of plastics and is generally used to assess the rate of degradation. Three pieces of plastic were randomly selected after co-culture with the strains removed from the surface biofilm with 2% SDS solution and dried. The changes in polymer structure of polymers with the bacterial incubation were analyzed using attenuated total reflection Fourier-transform infrared (ATR-FTIR) spectroscopy. Spectra from 4,000 to 400 cm^–1^ were acquired using the software. Images were acquired with a spectral resolution of 4 cm^–1^ and an average of 32 scans. The spectral data were water vapor compensation, baseline correction and smoothing according to the plastic identification protocol. All measured ATR-FTIR spectra were compared with spectra from a commercial library (Cross Sections Wizard, Polymer Laminated Films).

During degradation, the formation or disappearance of carbonyl groups and methyl groups were monitored (Sudhakar et al., 2007; Sudhakar et al., 2008). The carbonyl index measures the concentration of carbonyl groups and the double bond index measures the concentration of double bonds (Albertsson et al., 1987; Samat et al., 2023). In addition, the hydroxyl index measures the degree of oxidation of the plastic and is commonly used in the evaluation of degradation rates (Tang et al., 2019). It is assumed that no change in methyl groups occurs during degradation (Jeyakumar et al., 2013). The keto carbonyl, ester carbonyl, double bond (vinyl) and hydroxyl index of plastics were measured by FTIR spectroscopy using the following equation (Artham et al., 2009; Samat et al., 2023):

Keto carbonyl index = I_1715_/I_1465_
Ester carbonyl index = I_1740_/I_1465_
Double bond index = I_1640_/I_1465_
Hydroxyl index = I_3435_/I_1465_

Where relative intensities at 1,715, 1,740, 1,640, 3,435, and 1,465 cm^–1^ correspond to keto carbonyl, ester, double bond (vinyl index), hydroxyl index, and methylene bands, respectively (Rajakumar et al., 2009; Tang et al., 2019).

### 2.7 Scanning electron microscopy (SEM) of samples

After co-culture of bacteria and plastic particles, the morphology of the degraded samples was observed with SEM (FEI Quanta 250 FEG). The samples were sputtered with gold and palladium layers at 15 mA for 15∼30 s at 150 kPa argon gas and visualized using SEM at a maximum magnification of 10,000 × .

### 2.8 Examination of changes in angle of surface

After the end of the experimental cycle, the biofilm on the surface of PP and PS was washed with 2% SDS, washed three times and dried, and then the hydrophobicity change of the plastic surfaces was analyzed by contact angle. Firstly, the granules were fixed on double-sided tape, the contact angle was measured using a surface tensiometer (Rame-hart 500, American), and 4 μL of water was dropped onto the surface of the pellets at a rate of 2 μL/s. Each particle was measured three times and averaged for comparative analysis (Gu, 2003; Yang et al., 2015a; Liu et al., 2023).

### 2.9 Gas chromatography-mass spectrometry (GC-MS) analysis of degradation products released from polypropylene and polystyrene

Hydrocarbons were quantified by gas chromatography coupled with mass spectrometer (GC-MS). The supernatant was collected after filtration with filter paper, the supernatant was extracted with an equal volume of ethyl acetate, and the water was removed with Na_2_SO_4_ (Ruan et al., 2023). The sample passed through a 0.22 μm pore size membrane and was used as a GC-MS sample on a QP2020 NX instrument equipped with an HP-5MS column (100 m × 250 μm × 0.25 μm). The carrier gas is helium. The column temperature increased from 40 to 250°C at 10°C/min and maintained at 250°C for 4 min. The injector temperature is 300°C, the detector temperature is 260°C, and the scanning mass range is 33∼450 μm (Ghatge et al., 2022; Zampolli et al., 2023; Rong et al., 2024).

### 2.10 Statistical analyses

**FIGURE 1:**
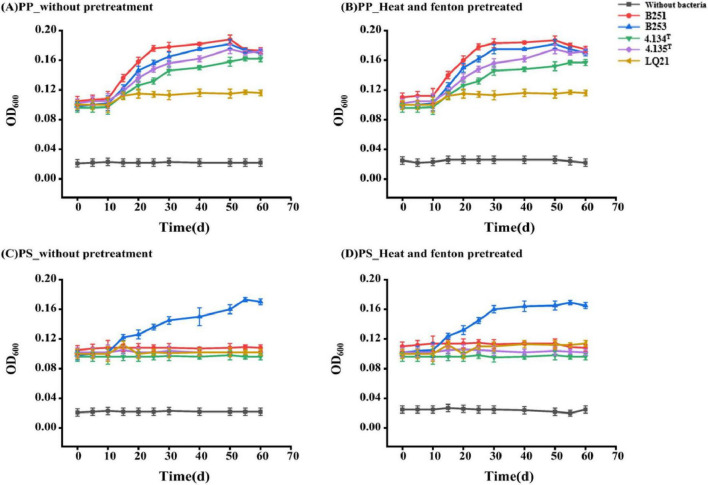
Growth curves of five *Gordonia* spp. strains using without-pretreatment polypropylene/polystyrene and pretreated polypropylene/polystyrene as the sole carbon source. **(A)** PP_without pretreatment **(B)** PP_Heat and fenton pretreated **(C)** PS_without pretreatment **(D)** PS_Heat and fenton pretreated.

**FIGURE 2:**
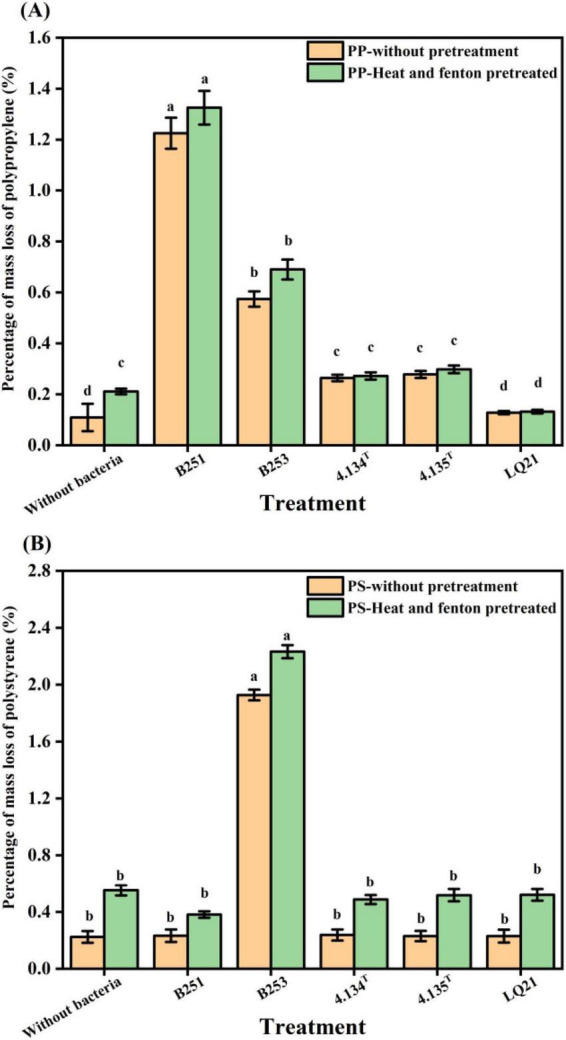
Weight loss rate of pretreated and untreated polypropylene and polystyrene pellets after 50 days of incubation with *Gordonia* spp. **(A)** Weight loss rate of untreated and pretreated polypropylene. **(B)** Weight loss rate of untreated and pretreated polystyrene. Error bars represent the standard error of the average of the three replicate readings of the percentage weight loss. Different lower-case letters indicate a statistically significant difference between the treatments, and data were statistically analyzed using SPSS one-way ANOVA and LSD *post-hoc* test, *p* < 0.05.

All experiments were conducted in triplicate and standard deviations are represented as error lines in Figures 1, 2. The mean variables and standard deviation were analyzed by Statistical Program for Social Sciences (SPSS 29.0.2.0, New York, IBM, United States). The data were statistically analyzed using SPSS one-way ANOVA and LSD *post hoc* tests, *P* < 0.05. Genomic data of *Gordonia polyisoprenivorans* strain B253 has been submitted to the NCBI database under submission number PRJNA1208225.

### 2.11 Genome sequence analysis

Genomic DNA integrity and purity were first evaluated as agarose gel electrophoresis. DNA samples were then broken into fragments of about 300 bp by Covaris ultrasonic crusher, and then the whole library was prepared through the steps of end repair, addition of A-tail, addition of sequencing splicer, purification, PCR amplification, etc. The library construction was initiated with quality control was performed using Qubit 2.0. After the library construction was completed, Qubit 2.0 was used for preliminary quantification, and the library was diluted to 2 ng/μL. Subsequently, the insert size of the library was detected using Agilent 2,100, and after the insert size met the expectation, the effective concentration of the library was accurately quantified using the qPCR method to ensure the quality of the library.

Following successful library validation, indexed libraries were normalized by effective concentration and pooled according to targeted downstream sequencing output, followed by Illumina HiSeq sequencing. Post-sequencing, raw reads were processed using Trimmomatic for quality control.

## 3 Results

### 3.1 Growth of *Gordonia* spp. with PP or PS as the sole carbon source

To study the growth of strains of *Gordonia* spp. when PP and PS particles were used as the sole carbon source, respectively, we selected five strains for growth curve measurement. As shown in Figure 1, the strains grew much slower with PP and PS as the only carbon source than those in nutrient medium (data was not shown).

During the initial 10 days of cultivation, minimal fluctuations in OD_600_ were observed, indicative of a delayed adaptive response of the strains to the environmental changes. After this lag phase, the strains gradually entered the logarithmic growth phase. Both *Gordonia polyisoprenivorans* B251 and *Gordonia polyisoprenivorans* B253 exhibited the ability to utilize polypropylene as the sole carbon source, with significantly enhanced growth rate when PP-pretreated was supplied compared to the control. *Gordonia hydrophobica* 4.134*^T^* and *Gordonia humi* 4.135*^T^* displayed intermediate growth performance, whereas the OD_600_ of *Gordonia sihwensis* LQ21 changed before 10 days compared to the control group, it did not change much after 10 days. Subsequently, *G. polyisoprenivorans* B251 and *G. polyisoprenivorans* B253 maintained continuous growth, while other strains exhibited slower proliferation rates. Quantitative comparisons revealed that among the five *Gordonia* species tested, *G. polyisoprenivorans* strain B251 demonstrated the highest growth capacity on PP plastics, followed by B253. Notably, even though all *Gordonia* strains showed measurable growth on PP when compared to the control, *Gordonia polyisoprenivorans* strains consistently outperformed other *Gordonia* strains in growth kinetics. After 50 days of incubation, the OD_600_ values stabilized across all strains. Biodegradation efficiency of the five *Gordonia* strains was consistent after heat and fenton pretreatment of PP granules (Figures 1A,B).

When polystyrene was used as the sole carbon source, all strains exhibited minimal change in OD_600_ during the initial 10-day incubation period, indicating that the strains were adapting to the environmental changes. After this period, the strain *Gordonia polyisoprenivorans* B253 entered into the logarithmic growth phase. In contrast to the control (without added bacteria), *G. polyisoprenivorans* B253 demonstrated the ability to utilize PS particles as the sole carbon source, with enhanced growth observed when PS- pretreated was supplied compared to PS- unpretreated. Conversely, other strains showed no significant changes relative to the control, considering that an inability to metabolize PS as a sole carbon substrate. Notably, *G. polyisoprenivorans* B253 was the only strain capable of proliferating with PS particles, with no measurable growth detected in other strains (Figures 1C,D). Pretreatment of PS using the same method applied to PP also exhibited enhanced growth rate, confirming the specificity of *G. polyisoprenivorans* B253 for aromatic polymer utilization under the tested conditions.

### 3.2 Mass loss of polypropylene and polystyrene treated by strains

After 50 days of incubation, medium with inoculated strains appeared turbid, whereas uninoculated controls remained optically clear, reflecting differential microbial proliferation. We quantified the degradation efficiency of five strains of *Gordonia* spp. on two plastic substrates by measuring the weight loss of polypropylene and polystyrene plastics.

The percentages of mass loss of polypropylene and polystyrene reflected the degradation efficiency to some extent, confirming the degradation of polypropylene and polystyrene particles by the strains (Figure 2). After 50 days of incubation, there were significant differences in the weight loss rate of PP particles treated with *Gordonia polyisoprenivorans* strain B251 and *Gordonia polyisoprenivorans* strain B253 compared to the uninoculated control. The percentages of mass loss of PP treated by strain *Gordonia polyisoprenivorans* B251 and *Gordonia polyisoprenivorans* B253 were 1.225 ± 0.061% and 0.574 ± 0.030%, respectively (Figure 2A). This result shows that these two strains have the ability to utilize PP as a sole carbon source for growth. Among the treatments, PS pellets inoculated with *G. polyisoprenivorans* B253 displayed the most pronounced weight loss rate when compared to both the control and other *Gordonia* spp. strains. After the treated of *Gordonia polyisoprenivorans* B253, the percentage of mass loss in PS was 1.927 ± 0.038%. These results suggest the strain B253 is also capable of utilizing PS as a sole carbon substrate for metabolic activity and proliferation (Figure 2B).

### 3.3 Changes of functional group on the surface of polypropylene and polystyrene treated with *Gordonia* spp.

**FIGURE 3:**
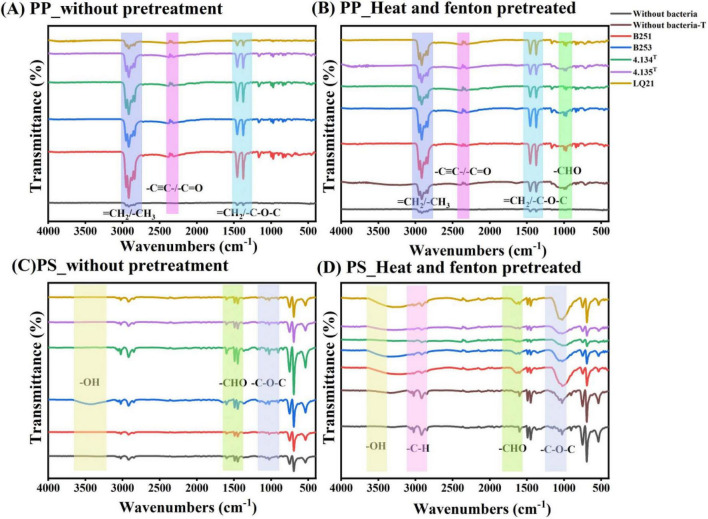
Attenuated total reflections Fourier transform infrared spectroscopy of polypropylene and polystyrene. **(A)** FTIR microscopy on PP pellets treated with *Gordonia* spp. **(B)** FTIR microscopy on pretreated PP pellets treated with *Gordonia* spp. **(C)** FTIR microscopy on PS pellets treated with *Gordonia* spp. **(D)** FTIR microscopy on pretreated PS pellets treated with *Gordonia* spp.

FTIR is a powerful approach to monitor chemical changes during the degradation of plastics. Based on the results of FTIR spectroscopy, we can see that the surfaces of the plastic particles co-cultured with the *Gordonia* strains all show changes in functional group. The PP surface showed changes in the C≡C triple bond or carbonyl C = O (2,348 cm^–1^), the C-H stretching vibration of the saturated hydrocarbons (2,979 cm^–1^), and the ether group of the oxygen-containing functional groups (1,359 cm^–1^). The appearance of these peaks compared to the control without bacterial treatment indicates the ability of *Gordonia* spp. to oxidize polypropylene and generate oxygen functional groups (Figure 3A). Stretching of ether groups, carbon-carbon triple bonds as well as carbon-hydrogen bonds were also present on the surface of the pretreated PP with the appearance of aldehyde groups (1,015 cm^–1^) compared to the control without bacterial treatment or pretreatment of the PP (Figure 3B). Ether groups (1,256 cm^–1^), alcohols (1,572 cm^–1^) containing oxygen functional groups were present on the PS surface. The presence of a hydroxyl peak (3,235 cm^–1^) after treatment of PS with *Gordonia polyisoprenivorans* strain B253 compared to the control without bacterial treatment indicates that *Gordonia polyisoprenivorans* strain B253 can oxidize polystyrene and generate oxygen functional groups (Figure 3C). There was a stretching of carbon-hydrogen bonds on the surface of the pretreated PS compared to the control, which was not changed by *Gordonia* spp. or pretreatment (Figure 3D).

### 3.4 The surface morphology of polypropylene and polystyrene changed by the treatment of *Gordonia* spp.

**FIGURE 4:**
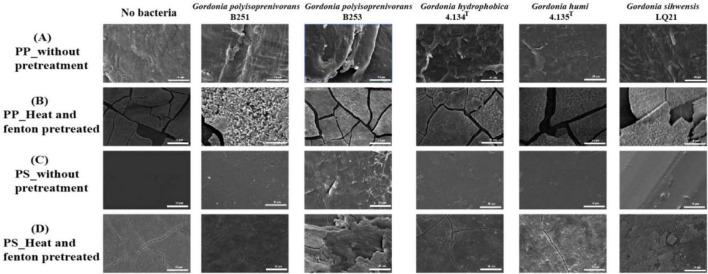
Scanning electron micrographs of PP and PS (10,000 × ). After pretreatment, the surface morphology of the plastic changed significantly. **(A)** PP_without pretreatment **(B)** PP_Heat and fenton pretreated **(C)** PS_without pretreatment **(D)** PS_Heat and fenton pretreated.

The severity of PP and PS biodeterioration was further validated using scanning electron microscopy (SEM) to observe the surface morphological changes. As shown in Figure 4, the surface of PP in the control group (without bacteria) was smooth. The surface morphology of the plastic co-incubated with the bacterial strains changed significantly and cracks appeared. More cracks were observed on the surface of PP samples cultured with *Gordonia polyisoprenivorans* strains compared to other *Gordonia* spp. with or without pretreatment (Figures 4A,B). Similarly, the same phenomenon was also observed in the treatment of PS. *Gordonia polyisoprenivorans* strain B253 exhibits a rougher surface and the appearance of cracks in PS treatment, suggesting that this strain is the most likely to colonize, when compared to the control group (no bacterial treatment) and the other treatment groups in Figures 4C,D. The various cracks that form on the surface of plastics are the result of microbial action. After heat and Fenton pretreatment, the plastic surface significantly changed and cracks appeared on the surface.

### 3.5 Confirmation of biodegradation of polypropylene and polystyrene by changes in surface physical properties

**FIGURE 5:**
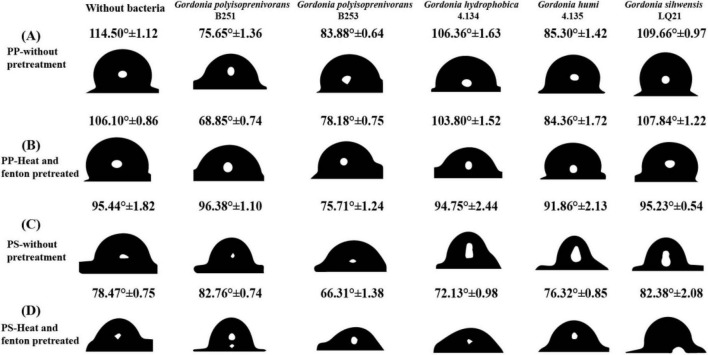
Changes in the contact angle between water droplets and PP/PS surfaces. **(A)** PP_without pretreatment **(B)** PP_Heat and fenton pretreated **(C)** PS_without pretreatment **(D)** PS_Heat and fenton pretreated. The contact angle formed by the water droplets with the PP surface decreased after the addition of *Gordonia* spp. compared to the control (without bacteria) and the contact angle was also reduced when the plastic was pretreated. Each profile was marked with a numerical value that indicated the magnitude of the water contact angle. Among them, *Gordonia polyisoprenivorans* B251 had the best degradation effect on PP, followed by *Gordonia polyisoprenivorans* B253. *Gordonia polyisoprenivorans* B253 had the best degradation effect on PS.

The hydrophobicity changes of PP and PS surfaces degraded by *Gordonia* spp. were characterized by water contact angle. Following biofilm removal from the polymer surfaces using 2% SDS and air-drying, the contact angles of PP and PS surfaces cultured with colonies were lower than those of uncultured controls (average = 114.50°, 95.44°, respectively) (*n* = 3, *t*-test, *P* < 0.05) (Figures 5A,C). The average values of the contact angles between the pretreated plastic surfaces and water were 106.10 and 78.47° (Figures 5B,D), which were greater than those of the co-incubation with bacteria.

### 3.6 Degradation products released from polypropylene and polystyrene

**FIGURE 6:**
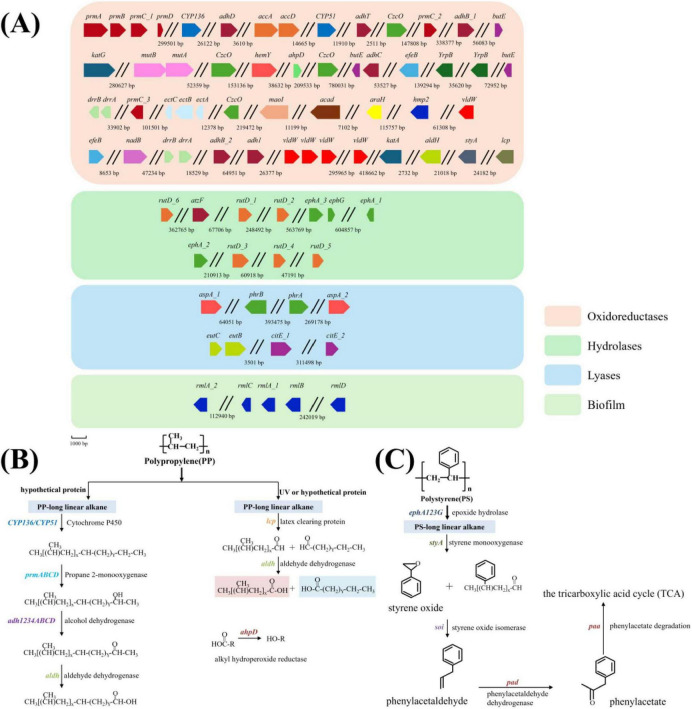
Functional genes of *Gordonia polyisoprenivorans* B253 and predicted degradation pathways. **(A)** The above genes were found in the gene sketch, including oxidoreductases, hydrolases, lyases and genes related to biofilm. **(B)** Predicted degradation pathway of polypropylene. **(C)** Predicted degradation pathway of polystyrene.

The growth of five *Gordonia* spp. isolates in inorganic salt liquid medium containing polypropylene and polystyrene as carbon sources was recorded using GC-MS analysis for 50 days with continuous shaking at 28°C. A total of 23 by-products were recorded (Supplementary Table S1). A selection of compounds related to degradation were screened from the table and plotted in the possible degradation pathways based on their chemical formulas (Figures 6B,C). The compounds of PP degradation identified are Z-7-Hexadecenal, 2-Methyl-6-methylene-octa-1,7-dien-3-ol, Hexadecanoic acid, 2-Butoxyethyl acetate and so on. The compounds of PS degradation identified are Dibutyl phthalate, 2,5-Di-tert-butylhydroquinone, 2-Cyclododecenol and so on (Supplementary Table S1).

### 3.7 Genomic annotation of *Gordonia polyisoprenivorans* strain B253

The incubation study indicated that the *Gordonia polyisoprenivorans* strain B253 possess the capability to degrade both PP and PS. To predict the functional genes involved in PP/PS degradation, we sequenced its genome, yielding approximately 1.69 GB of high-quality clean data. The statistical analysis of the three categories of Gene Ontology (GO) database revealed that the presence of three types of genes classified under cell components, with a predominant representation of genes associated with cellular structure. Additionally, KEGG annotation identified six major categories of metabolic pathways, cellular processes, environmental information processing, genetic information processing, human diseases, metabolism, and organismal systems with respective subclasses of 3, 2, 4, 4, 12, and 3. These pathways are primarily involved in cell communities-prokaryotes, membrane transport, amino acid metabolism, carbohydrate metabolism, and the metabolism of terpenes and polyketides. Collectively, these findings highlight the extensive polymer degradation potential of *G. polyisoprenivorans* B253.

Based on the genome annotation, numerous genes related to degradation were identified (Figure 6A and Supplementary Table S2), including latex clearing protein (*lcp*), Cytochrome P450 (*CYP136*), alcohol dehydrogenase (*adhABC*), flavin-containing monooxygenase (*CzcO*), styrene monooxygenase (*styA*) and so on. The predicted pathways of degradation of PP and PS by *G. polyisoprenivorans* strain B253 were derived from alignment with public database and relevant literature (Figures 6B,C). The genome of strain contains one gene encoding a latex scavenging protein, four genes encoding propane monooxygenase, two genes encoding cytochrome P450 monooxygenases, nine genes encoding ethanol dehydrogenase, one gene encoding amine oxidase, one gene encoding styrene monooxygenase, four genes encoding epoxide hydrolases, and one gene encoding alkyl hydroperoxide reductase. The genes demonstrate potential functionality in the degradation of both PP and PS.

Comparative analysis on the genome of *Gordonia polyisoprenivorans* B251 and *Gordonia polyisoprenivorans* B253 revealed that the genome size of B251 is 6,280,027 bp, while that of B253 is about 6,247,498 bp. The GC content of B251 and B253 genome are 66.99 and 67.01%, respectively. Both strains possess genes involved in polymer degradation, such as latex clearing protein (*lcp*), cytochrome P450 (*CYP*), aldehyde dehydrogenase (*aldh*), alkyl hydroperoxide reductase (*ahpD*) and so on (Supplementary Table S3).

## 4 Discussion

### 4.1 The growth and degradation ability of strains using PP/PS as sole carbon sources

This study aimed to investigate the ability to *Gordonia* spp. to degrade other C-C bonded plastics, especially polypropylene and polystyrene, and to determine whether pretreatment can enhance the degradation of plastics by these strains. We adopted a methodology similar to that used in previous studies (Liu et al., 2023; Samat et al., 2023; Li, 2024). In our study, growth curves were measured for five strains using PP or PS as the sole carbon sources. The OD_600_ values measurement revealed that *Gordonia polyisoprenivorans* B251 and *Gordonia polyisoprenivorans* B253 were capable of utilizing with PP as the sole carbon source, outperforming the other strains, which exhibited lower growth rate. Additionally, *Gordonia polyisoprenivorans* B253 was able to grow with PS as the sole carbon source and showed the highest growth rate compared to the control.

The pretreatment of PP and PS with heat and Fenton’s reagent, resulted in increased OD_600_ values, indicating that these pretreatments positively influence plastic degradation and promotes the growth of the associated strains. This effect may be attributed to thermal pretreatment, which disrupts the crystalline structyre of the plastic at elevated temperatures (Thomsen et al., 2023; Ciuffi et al., 2024). Additionally, Fenton’s treatment may decompose molecular chains through oxidation (Ghatge et al., 2022; Ciuffi et al., 2024), thereby reducing crystallinity (Samat et al., 2023) and facilitating the utilization of the plastic as a carbon source by the strains. Plastics undergo pyrolysis at high temperatures, which may release a large amount of volatile substances. Excessive heating can also cause dense oxide layers on the surface of plastics, which are difficult for microorganisms to use as a nutrient source. Incompletely decomposed H_2_O_2_ has strong oxidation properties, which will inhibit the activity of enzymes in microorganisms and may seriously damage the cellular structure.

In this study, we first employed weight loss measurement to characterize the degradation of PP and PS by *Gordonia* spp. The results indicated that polypropylene treated with *G. polyisoprenivorans* B251 exhibited the highest weight loss, followed by *G. polyisoprenivorans* B253. Additionally, polystyrene treated with *G. polyisoprenivorans* B253 also demonstrated the highest weight loss. The degradation rates observed for these strains were comparable to, and in some cases much higher than those reported for marine isolates in previous studies. For instance, *Rhodococcus ruber* C208 exhibited a massloss of only 0.8% in PS films after 8 weeks of incubation (Mor and Sivan, 2008). Similarly, *Exiguobacterium* sp. YT2, isolated from the gut of mealworms, demonstrated 7.4% ± 0.4% mass loss in PS pieces (2,500 mg/L) over a 60-day incubation period (Yang et al., 2015a,b). Furthermore, the enrichment of mangrove EPS waste yielded three strains of *Gordonia*, specially *Gordonia* sp. ZN14R1, ZN15R9, and ZN17RX, which resulted in the percentage of mass losses of 4.69 ± 1.91%, 7.73 ± 2.65%, and 6.69 ± 1.14%, respectively (Liu et al., 2023).

In our study, FTIR analysis revealed that polypropylene treated with *G. polyisoprenivorans* B253 exhibited the formation of oxygenated functional groups (carbonyl bands and ether groups) and double carbon bonds. These findings suggest that oxidation or oxygenation occurred on the surface of polypropylene. In contrast, no significant changes were observed on the control sample, which may be due to the high surface tension of the plastic after the treatment of the strain. Regarding polystyrene, treatment with *G. polyisoprenivorans* B253 resulted in the presence of various oxygenated functional groups, such as hydroxyl, aldehyde and ether groups. The accelerated degradation of PP and PS by *Gordonia polyisoprenivorans* B253 was further supported by pretreatment, indicating that its ability to break down C-C bonds extends to carbon polymer degradation. These findings are consistent with previous biodegradation studies. Notably, the study reported that the ketocarbonyl index of heat-treated MFPP is high, in *Aspergillus terreus* and *Engyodontium album*, although lower than that of the heat-treated control, except after 60 days of incubation. In addition, the heat-treated control appeared to have a higher carbonyl index than the WPC control. These changes imply the formation of new functional groups, which alter the surface energy of the polymer (Artham et al., 2009; Samat et al., 2023). In the bacterially treated samples, it is known that the characteristic peaks representing the PS benzene ring are suppressed, the intensity of the characteristic peaks of PS decreases and the carbonyl peaks appear to increase (Liu et al., 2023). Isolates from gut of *Zophobas atratus* or *Tenebrio molitor* could form carbonyl groups (C = O) in the oxidation pathway during PS biodegradation (Kim et al., 2020; Lou et al., 2020; Yang et al., 2021).

In the present study, it was observed by scanning electron microscopy that the surface of polypropylene treated by *Gordonia* spp. strains showed significant changes as compared to the control. Cracks appeared on the surface of PS treated with *G. polyisoprenivorans* B253, whereas the other *Gordonia* spp. strains showed no significant changes. The surface of plastic pretreated became significantly irregular, which is consistent with the following results. For instance, the formation of biofilm on the surface of plastics is believed to favor biodegradation (Meera et al., 2022). Parallelly, the surface of PP samples pretreated with UV, heat and fenton showed various cracks and irregularities. Therefore, it can be proved that pretreatment changes the surface morphology of the plastic (Samat et al., 2023). Sivan et al. used fluorescence microscopy to examine the viability of cells in biofilms grown on PS membranes (Sivan et al., 2006). Scanning electron microscopy observations revealed that all PS-degrading strains developed dense biofilms on the surfaces of PS, which became tightly packed following a 15-day incubation period. After 15 days of incubation, these biofilms were tightly attached to the PS surface, resulting in depression or even fracture of the plastic surface. At 30 days, the PS surface was severely eroded, and depressions were formed (Liu et al., 2023).

The water contact angle on the surface of PP plastic treated by *Gordonia polyisoprenivorans* B251 was the smallest, followed by *Gordonia polyisoprenivorans* B253. The water contact angle of the PS plastic surface treated by *Gordonia polyisoprenivorans* B253 was the smallest, and the rest of the *Gordonia* spp. strains did not differ much from the control. This result suggests that the two strains of *Gordonia polyisoprenivorans* reduced the hydrophobicity of PP and PS, which was attributed to the insertion of oxygen during the oxidation process, resulting in more and stronger polar interactions with water. After pretreatment, the water contact angle can be reduced, resulting in a more hydrophilic plastic surface. In the colonies grown on the PS surface, the water droplets formed a smaller contact angle with the PS surface than the control. The results suggest that *Gordonia* sp. and *Novosphingobium* sp. convert hydrophobicity to hydrophilicity during the degradation of PS (Liu et al., 2023).

GC-MS analysis of the degradation products revealed that the compounds identified from PP degradation included Z-7-Hexadecenal, 2-Methyl-6-methylene-octa-1,7-dien-3-ol, Hexadecanoic acid, 2-Butoxyethyl acetate, among others. For PS degradation, identified compounds included Dibutyl phthalate, 2,5-Di-tert-butylhydroquinone, 2-Cyclododecenol and so on (Supplementary Table S1). It has been reported that various compounds in the degradation of PE and PP microplastics were identified after treatment with high-efficiency composite bacteria, including 1,1’-oxobis-2-propanol, DL-propionamide methyl ether, 2,2,5-trimethyl-3,4-hexanedione, N, N’-dimethyl-1,3-propanediamine, tetrahydrofurfuryl n-butyrate and 3,3-dimethylhexane in PP group (Li, 2024). Additionally, it has been also reported that GC-MS analysis identified small organic acids, including acetic acid and butyric acid, as the major metabolites released from sulfonated PE (SPE), in which, 21 times more acetic acid (4.78 mM) and 17 times more butyric acid (0.17 mM) were released from SPE than from free GO_*X*_, that released 0.22 mM and 0.01 mM of acetic acid and butyric acid, respectively, after 6 h of reaction with TiO_2_-GO_*X*_ under UV radiation. (Ghatge et al., 2022).

In current study, the effect of degrading PP and PS was analyzed from multiple perspectives, including chemical composition and surface morphology. Through various characterization methods, the results demonstrated that *Gordonia polyisoprenivorans* B253 could induce changes in the chemical structure of these plastics, effectively confirming its degradation ability for PP and PS. This study provides a new basis for understanding plastic biodegradation.

### 4.2 Potential ability of *Gordonia* in polymer degradation and degradation pathway analysis

Polypropylene and polystyrene are synthesized though the polymerization of propylene and styrene, respectively. Our experimental results indicate that *Gordonia polyisoprenivorans* B253 can degrade both polypropylene and polystyrene. This suggests that *Gordonia polyisoprenivorans* B253 may possess functional genes related to the degradation of polymers. Genome analysis reveals that certain genes are associated with the degradation of propylene and styrene, while other genes are related to the breakdown of components found within resins. Identified functional genes include oxidoreductases, hydrolases, lyases and genes related to biofilms (Figure 6A and Supplementary Table S2). Combined with comparative analysis of the pathway diagrams, it was evident that metabolism involves two metabolic processes, namely lipid metabolism and terpene and polyketide metabolism (Figures 6B,C). Integrating the annotation results published reports, we identified 11 potential functional enzymes that may have functions in degrading plastics. Consequently, we have predicted the metabolic pathways of *G. polyisoprenivorans* B253 for degrading PP and PS. The degradation of polyethylene (PE) has been shown to yield smaller products, such as linear fatty alcohols and alkanoic acids, through on a cascade enzymatic reaction involving intrachain hydroxylation, catalyzed by a hypothetical P450 enzyme with an engineered active site (Yeom et al., 2022). The degradation pathway of PS primarily involves the breakdown of styrene monomer (Liu et al., 2021). Additionally, specific microplastic enzymes isolated from microorganisms, the mechanisms by which various enzymes degrade microplastics, and the types of microplastics for which the degradation mechanisms are not yet known were described (Othman et al., 2021). Recent studies collectively predicted three metabolic pathways of polystyrene based on metabolite content and pathways (Quan et al., 2023).

The genome of *Gordonia polyisoprenivorans* B251 and *Gordonia polyisoprenivorans* B253 contains genes that encode a variety of essential enzymes, including oxidoreductases, hydrolases and monooxygenases. These genes are crucial for producing functional enzymes that catalyze the breakdown of polymer chain by specifically targeting ester bonds, aromatic rings, or carbon-carbon double bonds within the polymer backbone. The synergistic regulation of gene expression and the degradation pathway highlights the potential of *Gordonia polyisoprenivorans* B253 to degrade polymers such as polypropylene and polystyrene.

## 5 Conclusion

This study analyzed the growth and degradation processes of plastic-degrading microorganisms using weight loss rate, Fourier infrared spectroscopy, scanning electron microscopy, and contact angle with water. By comparing data from the experimental group (*G. polyisoprenivorans* strain B253 treated PP and PS) with the control group (without *G. polyisoprenivorans* B253), we determined that treatments with or without heat and fenton pretreatment exhibited enhanced degradation of PP and PS when exposed to *G. polyisoprenivorans* B253. Notably, the promotion effect of pretreatment was stronger when the microorganism was applied. Our findings indicate that effective plastic degradation of the plastic-degrading microorganism *G. polyisoprenivorans* strain B253 required pretreatment of the plastics. On the one hand, this indicates the low degradation efficiency of plastics. On the other hand, the important function of physicochemical treatments is improving the biodegradability of plastics.

Genomic analysis and functional annotation of *G. polyisoprenivorans* B253 revealed that it has gene functions for degrading polypropylene and polystyrene granules made from polymerized resins. To address the application prospects of *G. polyisoprenivorans* B253 in PP and PS degradation, we optimize the catalytic efficiency and substrate specificity of key enzymes by analyzing the regulatory mechanism of gene expression, and promote the engineering application from laboratory degradation to actual plastic waste treatment, so as to provide a sustainable solution for the biodegradation of “white pollutants.” In the future, alternative pretreatment methods can be employed to investigate various types of plastics, and strains may be enhanced through genetic engineering. Additionally, the development of cost-effective pretreatment technology will facilitate the acceleration of the industrialization process of microorganism based biodegradation. The integration of plastic degradation technology with microbial strains represents a crucial approach to addressing “white pollution.” Moreover, it can foster a global transition toward a circular economy by leveraging the synergistic benefits across environmental, economic and social dimensions. This integration holds significant practical significance and strategic value.

## Data Availability

The original contributions presented in the study are publicly available. This data can be found here: https://www.ncbi.nlm.nih.gov, accession number PRJNA1208225.

## Footnotes

1
http://www.ncbi.nlm.nih.gov/BLAST/
