# Supplementary material for: Biodegradation potential of Gordonia spp. on polypropylene and polystyrene: enhanced degradation through pretreatment
Source: Front Microbiol. 2025 Jul 25;16:1621498. doi: 10.3389/fmicb.2025.1621498 (PMC12332524; doi:10.3389/fmicb.2025.1621498)
Supplement: Supplementary file 1 [file Supplementary_file_1.docx]

**Supplemental Material**

**Biodegradation potential of *Gordonia* spp. on polypropylene and polystyrene and enhanced degradation by pretreatment**

Yan Zhu^a^, Hongzhe Wang^a, b^, Jing Bai^c^, Yanjie Qi^a^, Dongfei Han^a^*

^a^ School of Environmental Science and Engineering, Suzhou University of Science and Technology, Suzhou 215009, China.

^b^ Key Laboratory of Soil Environment and Pollution Remediation, Institute of Soil Science, Chinese Academy of Sciences, Nanjing 210008, China.

^c^ School of Chemistry and Life Sciences, Suzhou University of Science and Technology, Suzhou 215009, China.

* **The corresponding author:**

Dongfei Han

dongfeihan@usts.edu.cn

**Supplemental Tables (pages 2-9)**

**Supplemental Tables**

**Table S1.** GC-MS analysis of degradation products of PP and PS plastics

**Table S2.** Genes that may be involved in the degradation of PP and PS in the genome of *Gordonia polyisoprenivorans* B253

**Table S3.** The genomic information comparison of *Gordonia polyisoprenivorans* B251 and *Gordonia polyisoprenivorans* B253

**Table S1** GC-MS analysis of degradation products of PP and PS plastics

| **Name of the compound** | | **Treatment** | | | | | | | | | | | | |
| --- | --- | --- | --- | --- | --- | --- | --- | --- | --- | --- | --- | --- | --- | --- |
|  |  | **Chemical formula** | **Without pretreatment** | | | | | | **Heat and fenton pretreated** | | | | | |
|  |  |  | **Without**  **bacteria** | **B251** | **B253** | **4.134** | **4.135** | **LQ21** | **Without**  **bacteria** | **B251** | **B253** | **4.134** | **4.135** | **LQ21** |
| Polypropylene degradation products | Octadecanoic acid,  ethenyl ester | C_20_H_38_O_2_ | √ | × | × | × | × | × | × | × | × | × | × | × |
|  | E-2-Methyl-3-tetradecen-1-ol acetate | C_17_H_32_O_2_ | × | × | × | × | × | × | √ | × | × | × | × | × |
|  | **Z-7-Hexadecenal** | C_16_H_30_O | × | √ | √ | × | √ | × | × | × | × | × | × | × |
|  | **2-Methyl-6-methylene-octa-1,7-dien-3-ol** | C_10_H_16_O | × | × | × | × | × | × | × | × | √ | × | × | × |
|  | 1H-limidazole-2-methanol,1-decyl | C_14_H_26_N_2_O | × | × | × | × | × | × | × | √ | √ | × | × | × |
|  | **2-Butoxyethyl acetate** | C_8_H_16_O_3_ | × | × | × | × | × | × | × | √ | × | × | × | × |
|  | **Hexadecanoic acid** | C_16_H_32_O_2_ | × | × | × | × | × | × | × | √ | √ | √ | √ | √ |
|  | **2-Myristynoyl-glycinamide** | C_10_H_16_O | × | × | × | × | × | × | × | √ | √ | × | × | × |
|  | **5-Hexen-3-ol, 3-methyl** | C_8_H_16_O | × | × | × | × | × | × | × | × | √ | × | × | × |
|  | 2-Naphthalene carboxylic acid | C_12_H_10_O_2_ | × | × | × | √ | √ | √ | × | × | × | × | × | × |
| Polystyrene degradation products | 9,12-Octadecadienoic acid (Z,Z)-,phenylmethyl ester | C_25_H_38_O_2_ | √ | √ | × | × | × | × | × | × | × | × | × | × |
|  | **9-octadecen-1-al** | C_18_H_34_O | × | × | √ | × | × | × | × | × | × | × | × | × |
|  | Benzyl linoleate | C_25_H_38_O_2_ | × | × | × | √ | × | √ | × | × | × | × | × | × |
|  | Benzonic acid, 2-formyl-4,6-dimethoxy-,8,8-dimethoxyoct-2-yl ester | C_20_H_30_O_7_ | × | × | × | × | √ | × | × | × | × | × | × | × |
|  | 9,9-Dimethoxybicyclo[3.3.1]nona-2,4-dione | C_11_H_16_O_4_ | × | × | × | × | × | × | √ | √ | × | × | √ | √ |
|  | **2,5-Di-tert-butylhydroquinone** | C_14_H_22_O_2_ | × | × | × | × | × | × | × | × | √ | × | × | × |
|  | ***cis*-9,10-Epoxyoctadecan-1-ol** | C_18_H_36_O_2_ | × | × | × | × | × | × | × | × | √ | × | × | × |
|  | **2-Cyclododecenol** | C_12_H_22_O | × | × | × | × | × | × | × | × | √ | × | × | × |
|  | **Dibutyl phthalate** | C_16_H_22_O_4_ | × | × | × | × | × | × | × | × | √ | × | × | × |
|  | **trans-2-Decenoic acid** | C_10_H_18_O_2_ | × | × | × | × | × | × | × | × | √ | × | × | × |
|  | 18-Pentatriacontanone | C_35_H_70_O | × | × | × | × | × | × | √ | √ | × | × | × | × |
|  | **1-Cyclohexylnonene** | C_15_H_28_ | × | × | √ | × | × | × | × | × | × | × | × | × |
|  | Cyclobabital | C_12_H_16_N_2_O_3_ | × | × | × | × | × | × | × | × | √ | × | × | × |

Notes: "√" means to be detected, and "×" means not detected.

**Table S2** Genes that may be involved in the degradation of PP and PS in the genome of *Gordonia polyisoprenivorans* B253

| **Enzyme type** | **Protein** | **Gene names** | **Gene** | **Function** |
| --- | --- | --- | --- | --- |
| Oxidoreductases | **Rubber oxygenase/Cell wall biosynthesis protein** | OCMCINGN_04810 | *lcp* | When the double bonds in the rubber are broken, a mixture of C_20_, C_25_, C_30_ and oligomeric isoprene with ketone and aldehyde groups at the end is produced |
|  | **Propane 2-monooxygenase** | OCMCINGN_00013,OCMCINGN_00014,  OCMCINGN_00015,OCMCINGN_00016,  OCMCINGN_00430,OCMCINGN_02005,  OCMCINGN_03173,OCMCINGN_04601 | *prmA*, *prmB*, *prmC*, *prmD* | Hydroxylation of mid-terminal carbon |
|  | **cytochrome P450** | OCMCINGN_00245,OCMCINGN_00288 | *CYP136*, *CYP51* | Hydroxylation of C–H, epoxidation of C=C, dealkylation of O-, N- and S-, aromatic coupling and cleavage of C-C |
|  | **alcohol dehydrogenase** | OCMCINGN_05086,OCMCINGN_00743, OCMCINGN_02133,OCMCINGN_00288, OCMCINGN_00267,OCMCINGN_00301, OCMCINGN_02183 | *adhA*, *adhB*, *adhC*, *adhD, adhT, adh1, adh2, adh3, adh4* | Dehydrogenation of hydroxyl groups to carbonyl or aldehyde groups |
|  | NAD(P)H-dependent flavin oxidoreductase | OCMCINGN_00867,OCMCINGN_00901 | *YrpB* | NA |
|  | Protoporphyrinogen oxidase | OCMCINGN_01677 | *hemY* | Synthesis of ferrous heme and chlorophyll |
|  | flavin-containing monooxygenase | OCMCINGN_00305,OCMCINGN_01529, OCMCINGN_01902 | *CzcO* | Oxygenase, which adds an oxygen atom to the substrate, involved in various of metabolic pathways in animals and plants |

**Table S2 continued**

| **Enzyme type** | **Protein** | **Gene names** | **Gene** | **Function** |
| --- | --- | --- | --- | --- |
| Oxidoreductases | Daunorubicin/doxorubicin resistance ATP-binding protein | OCMCINGN_02045,OCMCINGN_02116 | *drrA* | NA |
|  | Daunorubicin/doxorubicin resistance ABC transporter permease protein | OCMCINGN_02046,OCMCINGN_02114 | *drrB* | NA |
|  | putative methylmalonyl-CoA mutase | OCMCINGN_01485,OCMCINGN_01486 | *mutA, mutB* | Related to the synthesis of pyruvate and amino acids |
|  | Validamycin A dioxygenase | OCMCINGN_02208,OCMCINGN_02209  OCMCINGN_02210,OCMCINGN_02212  OCMCINGN_02489 | *vldW* | NA |
|  | L-arabinose transport system permease protein | OCMCINGN_02662 | *araH* | Proteins involved in arabinose transport |
|  | **Primary amine oxidase** | OCMCINGN_02682 | *maoI* | Synthesis of 4-hydroxyphenylacetaldehyde |
|  | peroxidase | OCMCINGN_02864,OCMCINGN_02063 | *efeB* | Splitting water produces hydrogen peroxide, which splits oxygen into hydroxide ions |
|  | **aldehyde dehydrogenase** | OCMCINGN_05509 | *aldh* | Converts aldehydes with oxygen in water molecules into carboxylic acids |
|  | **alkyl hydroperoxide reductase** | OCMCINGN_01711 | *ahpD* | Reduces the carboxyl group to the hydroxyl group |
|  | **styrene monooxygenase** | OCMCINGN_03631 | *styA* | Epoxy compounds are formed by oxidizing C=C on styrene |

**Table S2 continued**

| **Enzyme type** | **Protein** | **Gene names** | **Gene** | **Function** |
| --- | --- | --- | --- | --- |
| Oxidoreductases | **Catalase-peroxidase** | OCMCINGN_01259 | *katG* | It has high catalase activity, hydrogen peroxide as a donor, releases oxygen, and can act on peroxidase of a variety of organic matter, especially when ethanol is used as a hydrogen donor, it shows catalase and peroxidase activity belongs to catalase-peroxidase |
|  | **Catalase** | OCMCINGN_05267 | *katA* |  |
|  | glutathione peroxidase | OCMCINGN_00797,OCMCINGN_01085 | *btuE* | Catalyzes the decomposition of hydrogen peroxide to produce water, which is oxidized itself and reactivated by the glutathione reductase system |
|  | Dimethyl-sulfide monooxygenase | OCMCINGN_02841 | *dmoA* | NA |
|  | 3-aminobutyryl-CoA aminotransferase | OCMCINGN_03190 | *kat* | Catalyzes the reaction between 3-keto-5-aminocaproic acid and acetyl-CoA to generate acetoacetic acid and 3-aminobutyryl-CoA |
|  | transaminase | OCMCINGN_01917,OCMCINGN_01918  OCMCINGN_01919 | *ectA, ectB, ectC* | Catalyzes transamination between amino acids |
|  | L-aspartate oxidase | OCMCINGN_02070 | *nadB* | Catalyzes the oxidation of L-aspartic acid |

**Table S2 continued**

| **Enzyme type** | **Protein** | **Gene names** | **Gene** | **Function** |
| --- | --- | --- | --- | --- |
| Hydrolases | Allophanate hydrolase | OCMCINGN_00320 | *atzF* | Catalytic conversion of urea to ammonia |
|  | **Epoxide hydrolase** | OCMCINGN_01807,OCMCINGN_02345  OCMCINGN_03736OCMCINGN_02917 | *ephA1, ephA2, ephA3, ephG* | Catalyzes the hydrolysis of epoxides |
|  | Putative aminoacrylate hydrolase | OCMCINGN_00379,OCMCINGN_00604  OCMCINGN_02555,OCMCINGN_02612  OCMCINGN_02658,OCMCINGN_03273 | *rutD1, rutD2, rutD3, rutD4, rutD5, rutD6* | NA |
| Lyases | Aspartate ammonia | OCMCINGN_00152,OCMCINGN_00763 | *aspA_1, aspA_2* | NA |
|  | Citrate lyase subunit beta | OCMCINGN_02824,OCMCINGN_03136 | *citE_1, citE_2* | NA |
|  | Ethanolamine ammonia | OCMCINGN_05262,OCMCINGN_05263 | *eutB, eutC* | Catalyzes the rearrangement of ethanolamine |
|  | photolyase | OCMCINGN_00506,OCMCINGN_01366 | *phrA, phrB* | Enzymes involved in repairing DNA damage caused by ultraviolet radiation |
| Biofilm | Glucose-1-phosphate thymidylyltransferase | OCMCINGN_02405,OCMCINGN_03233 | *rmlA_1, rmlA_2* | NA |
|  | dTDP-4-dehydrorhamnose reductase | OCMCINGN_04544 | *rmlD* | Involved in the biosynthesis of lipopolysaccharide precursors |
|  | dTDP-glucose 4,6-dehydratase | OCMCINGN_02404 | *rmlB* | NA |
|  | dTDP-4-dehydrorhamnose 3,5-epimerase | OCMCINGN_02406 | *rmlC* | NA |

Note: "NA" means unknown.

**Table S3** The genomic information comparison of *Gordonia polyisoprenivorans* B251 and *Gordonia polyisoprenivorans* B253

| **Characteristic** | | **Value** | | |
| --- | --- | --- | --- | --- |
|  |  | ***G. polyisoprenivorans* B251** | ***G. polyisoprenivorans* B253** | |
| Size of genome (bp) | | 6,280,027 | 6,247,498 | |
| GC content (%) | | 66.99 | 67.01 | |
| No. of RNA genes | | 60 | 65 | |
| No. of tRNA genes | | 49 | 58 | |
| No. of rRNA genes | | 9 | 6 | |
| No. of mRNA genes | | 2 | 1 | |
| 16s Per. Ident | | 98.94% | | |
| ANI | | 99.99% | | |
| DDH | | 99.99% | | |
| Oxidoreductases | *lcp* | 1 | 2 | 100% |
|  | *CYP* | NA | 2 | NA |
|  | *aldh* | 1 | 1 | 76.63% |
|  | *ahpD* | 1 | 1 | 100% |
|  | *prm* | 4 | 4 | 100% |
|  | *adh* | 6 | 9 | 100% |
| Hydrolases | *eph* | 0 | 4 | NA |
|  | *acm* | 2 | 0 | NA |
| Lyases | *phrA* | 0 | 2 | NA |
| Biofilm | *rmlA* | 1 | 2 | 96.52% |
|  | *rmlB* | 1 | 1 | 99.70% |
|  | *rmlC* | 1 | 1 | 100% |
|  | *rmlD* | 1 | 1 | 100% |

Note: "NA" means unknown.
